# Urinary exosomal long noncoding RNAs serve as biomarkers for early detection of non-small cell lung cancer

**DOI:** 10.1042/BSR20210908

**Published:** 2021-10-12

**Authors:** Quan Lin, Danli Xie, Liangliang Pan, Yongliang Lou, Mengru Shi

**Affiliations:** 1 The First Affiliated Hospital of Wenzhou Medical University, Wenzhou, Zhejiang 325035, China; 2 Zhejiang Provincial Key Laboratory of Medical Genetics, Wenzhou, Zhejiang 325035, China; 3 Key Laboratory of Laboratory Medicine, Ministry of Education, Wenzhou, Zhejiang 325035, China; 4 School of Laboratory Medicine and Life Science, Wenzhou Medical University, Wenzhou, Zhejiang 325035, China

**Keywords:** lncRNA, exosome, urine, biomarker, NSCLC

## Abstract

**Objective:** Increasing the efficiency of early diagnosis using noninvasive biomarkers is crucial for enhancing the survival rate of lung cancer patients. We explore the differential expression of non-small cell lung cancer (NSCLC)-related long noncoding RNAs (lncRNAs) in urinary exosomes in NSCLC patients and normal controls to diagnose lung cancer.

**Methods:** A differential expression analysis between NSCLC patients and healthy controls was performed using microarrays. Gene ontology (GO) term and Kyoto Encyclopedia of Genes and Genomes (KEGG) pathway analyses were used to predict potential functions of lncRNAs in NSCLC. quantitative real-time PCR (QT-PCR) was used to verify microarray results.

**Results:** A total of 640 lncRNAs (70 up- and 570 down-regulated) were differentially expressed in NSCLC patients in comparison to healthy controls. Six lncRNAs were detected by QT-PCR. GO term and KEGG pathway analyses showed that differential lncRNAs were enriched in cellular component organization or biogenesis, as well as other biological processes and signaling pathways, such as the PI3K-AKT, FOXO, p53, and fatty acid biosynthesis.

**Conclusions:** The differential lncRNAs in urinary exosomes are potential diagnostic biomarkers of NSCLC. The lncRNAs enriched in specific pathways may be associated with tumor cell proliferation, tumor cell apoptosis, and the cell cycle involved in the pathogenesis of NSCLC.

## Introduction

Lung cancer is one of the three most commonly occurring malignancies on a global scale. Non-small cell lung cancer (NSCLC) accounts for 85% of lung cancer cases [1,2]. Despite considerable advances in the early diagnosis and treatment of early-stage lung cancer, the 5-year survival rate of NSCLC patients remains low, and early diagnosis of NSCLC is still a major challenge [3,4]. Therefore, the exploration of potential molecular targets for early detection or intervention/treatment of lung cancer is urgently needed to reduce lung cancer mortality [5,6].

Exosomes are microvesicles with diameters of 30–100 nm secreted by a large variety of cells from various biofluids that carry a repertoire of functional biomolecules, including genomic DNA, RNA, microRNA, long noncoding RNAs (lncRNAs), and protein [7,8]. Exosomes serve as potential biomarkers for early cancer detection [9]. The lung cancer-derived exosome represents the cell of origin in numerous aspects [4,10]. Hence, it must be intensively studied to better understand cell–cell communication and cancer proliferation.

LncRNAs are a group of noncoding RNAs with more than 200-nucleotide length [11,12]. Recently, numerous experimental and clinical evidence have shown that lncRNAs are involved in epigenetic regulation, remodeling of chromosomes, transcription, and post-transcriptional regulation and play important roles in tumorigenesis and tumor progression [13,14]. The reduction in several lncRNAs has been reported in various tumors, such as colorectal carcinoma, osteosarcoma, hepatocellular carcinoma, gastric cancer, bladder cancer, and lung cancer [15–19]. The correlation between lncRNAs and the pathogenesis of NSCLC has received significant attention [20]. To date, there have also been studies focusing on the analysis of lncRNAs in exosomes. Some studies have demonstrated that numerous lncRNAs in exosomes such as GAS5-AS1, lncRNA BX357664, HOTTIP, and MALAT1 have their potential role in the diagnosis, prognosis, and treatment of lung cancer patients [21–24]. In the study of Zhang et al. [24], exosomal *MALAT-1* was highly expressed in NSCLC patients. Exosomal lncRNA *MALAT-1* was demonstrated to be associated with the growth and proliferation of tumor cells. Dong et al. found that exosomal lncRNA*GAS5* was down-regulated in NSCLC patients [25]. However, the functions and mechanisms of lncRNAs in NSCLC have not been fully understood. In the present study, we compared several lncRNAs in the urinary exosome samples between NSCLC patients and healthy controls using microarrays to explore the differential expression of exosome lncRNAs and miRNAs. The results revealed the dysregulation of lncRNAs and miRNAs in exosomes from urine samples. We used the Gene ontology (GO) and the Kyoto Encyclopedia of Genes and Genomes (KEGG) analyses to evaluate the potential signaling pathway of differential expression lncRNAs and we then constructed the interaction network of lncRNAs–mRNA. Our data suggest that the specific lncRNAs and miRNAs in urinary exosomes can serve as noninvasive biomarkers for the diagnosis of early-stage NSCLC.

## Materials and methods

### Patients and samples

Twenty NSCLC patients (age [mean ± SD] = 64.60 ± 7.8 years) from the Department of Respiratory Medicine of the First Affiliated Hospital of Wenzhou Medical University were enrolled in the present study. All patients had confirmed pathological diagnosis according to the 2017 lung cancer staging system of the AJCC Cancer Staging Manual 8e [26]. The 20 control subjects were healthy volunteers. The clinical information on patients and controls is listed in Table 1.

**Table 1:** Clinical characteristics of NSCLC patients and healthy controls

Study groups	Number of samples	Parameters	
**NSCLC patients**	20	Gender	***n* (%)**
		Male	10 (50)
		Female	10 (50)
		Age (years) mean ± SD	64.60 ± 7.8
		Pathological type	***n* (%)**
		Squamous carcinoma	6 (30)
		Adenocarcinoma	14 (60)
		Clinical stage	***n* (%)**
		I + II stage	7 (35)
		III + IV stage	13 (65)
**Healthy controls**	20	Gender	***n* (%)**
		Male	10 (50)
		Female	10 (50)
		Age (years) mean ± SD	52.6 ± 11.09

Patients with the following conditions were excluded: (1) suffering from other rheumatological diseases; (2) having concomitant complications of severe heart, kidney, or liver disease; (3) having received prior therapy for NSCLC, including chemotherapy, chemoradiotherapy, biological/targeted therapy, or any investigational drug. All analyses were performed following relevant ethical guidelines and regulations. The research was approved by the Ethics Committee of The First Affiliated Hospital of Wenzhou Medical University. And the research has been carried out in accordance with the World Medical Association Declaration of Helsinki*. All study participants signed informed consent.

### Urine sample collection

A total of 200 ml first-morning urine samples was collected from each participant and mixed with 16.8 ml protease and phosphatase inhibitors (1.67 ml of 100 mmol/l NaN_3_, 2.5 ml of 10 mmol/l PMSF, 50 µl of 1 mmol/l Leupeptin for every 50-ml urine) immediately after collection. All samples were centrifuged at 1000×***g*** for 10 min at 4°C to remove cell pellets and then stored at −80°C until further use.

### Urine exosome isolation

The thawed urine samples were centrifuged at 17000×***g*** for 20 min at 4°C to remove whole cells, large membrane fragments, and other debris. The supernatants were ultracentrifuged at 200000×***g***, for 1 h at 4°C in the Beckman Qtima100 ultracentrifuge (Beckman, U.S.A.) to obtain sediments [27,28]. The sediments were resuspended in 0.01 mol/l of PBS solution.

### Negative staining by transmission electron microscopy

A total of 20 μl of exosome suspension was loaded on a 100 mesh sample-loaded copper mesh and left at room temperature for 1 min. Excess liquid was blotted with filter paper from the other side of the grid. Subsequently, the exosome sample was stained with 3% phosphotungstic acid for ∼10 min after air-drying. Samples were observed under transmission electron microscopy (TEM H-7500, Hitachi, Japan).

### Nanoparticle tracking analysis

The exosome hydrodynamic size and number were obtained by nanoparticle tracking analysis (NTA), performed by Nano Sight NS300 (Malvern Panalytical, Ltd.) equipped with rapid video capturing following the manufacturer’s instructions [29,30].

### Western blot

Total urinary exosome proteins were extracted using the RIPA lysis buffer (50 mmol/l Tris-HCl (pH 7.4), 150 mmol/l NaCl, 1% NP-40, 0.1% SDS, 1 mmol/l PMSF). Protein concentrations were measured by the BCA Protein Assay Kit (ab102536, Abcam). A Western blot was performed using 12% polyacrylamide/SDS gels (SDS/PAGE) in a Mini Trans-Blot module (Bio-Rad, U.S.A.) and transferred on to polyvinylidene fluoride (PVDF, GE) membranes. After blocking, the membranes were probed overnight with primary antibodies against CD63 (ab134045, Abcam, 1:1000), CD9 (ab263019, Abcam, 1:1000), and TSG101 (sc-7964, Santa Cruz, 1:1000). The membranes were washed three times with TBST the following day and incubated with HRP-conjugated anti-mouse IgG or HRP-conjugated anti-rabbit IgG (1:1500, Santa Cruz) as the secondary antibody at room temperature for 1 h. The signals were visualized with the enhanced chemiluminescence (ECL) kit (Thermo Scientific, U.S.A.) using the ChemiDoc MP Imaging System (Bio-Rad, U.S.A.) according to the manufacturer’s instructions.

### RNA extraction of exosome

The total RNA of exosomes was extracted using the TRIzol reagent (Invitrogen, Carlsbad, CA, U.S.A.), and the concentration and quality of RNA were determined by quantification on the Nanodrop 2000 (Thermo Scientific, U.S.A.). The RNA integrity was detected by Agilent Bioanalyzer 2100 (Agilent Technologies, U.S.A.). Total RNA was purified using a QIAGEN RNeasy Mini Kit (QIAGEN).

### Library construction and RNA sequencing using microarray hybridization

Total purified RNA from each sample was reverse transcribed into first-strand cDNA using an AffinityScript-RT kit and promoter primer (Agilent Technologies, U.S.A.). Subsequently, second-strand cDNA was generated using an antisense promoter (Agilent Technologies, U.S.A.). The double-stranded cDNA was used as a template for amplification with T7 RNA polymerase to create antisense cRNA.The cRNA was labeled with Cyanine-3 (Cy3) CTP, and then purified by a QIAGEN RNeasy mini kit (QIAGEN). Labeled cRNA was then fragmented and hybridized on to the LC Biotech human lncRNA microarray (Agilent). The microarrays were incubated for 17 h at 65°C in an Agilent hybridization oven. Following washing, the microarrays were scanned using the Agilent Scanner G5761A (Agilent Technologies, Inc.).

### Differential expression analysis of lncRNAs

The image data were processed using the Feature Extraction version 12.0.3.1 (Agilent Technologies). Raw data were normalized by the Quantile algorithm in Genespring (version 14.8, Agilent Technologies) software. The standardized data were filtered, and at least one set of 100% probes labeled ‘Detected’ from each set of samples was used for comparison was stored for subsequent analysis. Differential gene expression of the lncRNAs was considered significant for *P*≤0.05 obtained by the Student’s *t* test between groups. The clinical information on patients and controls is listed in Supplementary Table S1.

### Cluster analysis of differential lncRNA expression

The hierarchical clus0ter analysis of differentially expressed lncRNAs was performed using CLUSTER3.0, and the following volcano plot and heatmap analysis were performed.

### Validation by quantitative real-time PCR

Reverse transcription and quantitative real-time PCR (QT-PCR) were performed to confirm the difference in the expression of the six selected lncRNAs between NSCLC patients and the control group. **ChamQ™ Universal SYBR® qPCR Master Mix (Vazyme, China)** was used to perform QT-PCR on the CFX96 system (Bio*-*Rad Laboratories, Inc., Hercules, CA, U.S.A.). After normalization to U6, the relative expression level of lncRNA was calculated by the 2^−ΔΔ*C*_T_^ method. The primer sequences are listed in Table 2.

**Table 2:** Nucleotide sequence of primers used for verification of the differentially expressed lncRNAs in NSCLC patients

Gene name	Forward primer	Reverse primer
lnc-FRAT1-5	ACTGCTGCGAGGAGGAAAAT	GCCCTGGATGTGTGCTTTTC
lnc-SRY-11	TGACTTCTCAGGGCTGCAAG	TGAGGGTTCCAAGTTCACGG
lnc-RNASE13-1	TCAGCAGGGTTGGGAATGTC	CCAGCACCATCCCCTTCTTT
lnc-RP11-80A15.1.1-2	GCCAAGCCTGCTATCTCCTA	GGTGTCAATGTGGCTTTGGG
lnc-ARL6IP6-4	TCAACCTTGACTTCAGGGCC	AGACTTAACAATTGGCGCGC
lnc-DGKQ-1	GGCTTTACCAGGCCTTCTGT	TTCCTACACATGGTGCCCAC
U6	GCGCGTCGTGAAGCGTTC	GTGCAGGGTCCGAGGT

### Target gene prediction and functional analysis

LncRNA regulates the neighboring genes’ expression, which can be predicted based on the position within a range of 100 kb of its target gene of lncRNA and mRNA. To explore the function of lncRNAs, we first predicted the *cis* and *trans* target genes of lncRNAs.

Differentially expressed lncRNAs were then further analyzed with GO enrichment and KEGG pathway database to identify the involved enriched pathways of all differentially expressed genes. In GO enrichment analysis, there are three structured relationships of defined terms that describe gene product attributes, namely, the biological process, molecular function, and cellular component. The KEGG pathway analysis was carried out to reveal potential signaling clusters covering the differentially expressed genes. *P*<0.05 was considered statistically significant.

### Statistical analysis

All statistical analyses were performed using the GraphPad Prism 5.0 (GraphPad Software, La Jolla, CA, U.S.A.) and SPSS 26.0 software (SPSS, Chicago, IL, U.S.A.). Differences of each exosomal lncRNA between NSCLC patients and control samples were performed using the *t* test for paired data. *P*≤0.05 was considered statistically significant.

## Results

### Isolation and verification of urinary exosomes

**Figure 1:**
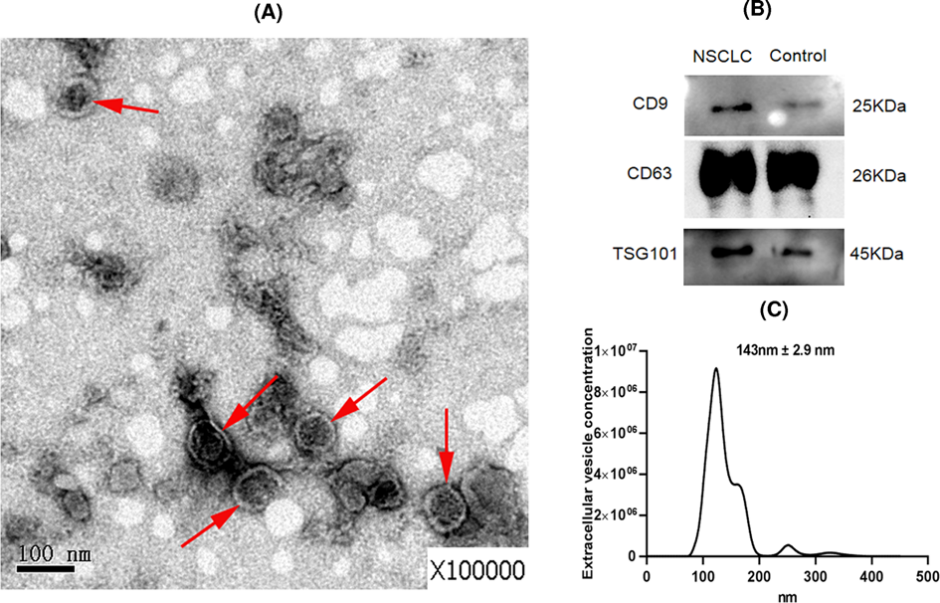
Characterization of urinary exosomes in NSCLC (**A**) Urinary exosomes visualized by transmission electron microscopy. Exosomes are indicated by red arrows. Scale bar = 100 nm. (**B**) Western blotting results for exosome-enriched proteins CD63, CD9, and Tsg101. (**C**) NTA result of exosomes.

We used ultracentrifugation to isolate urinary exosomes from NSCLC patients and healthy controls. Qualitative negative staining electron microscopy was used to verify whether the sediments were exosomes. In TEM analysis, isolated urinary exosome vesicles appeared as flattened spheres with diameters ranging from approximately 30 to 150 nm (Figure 1A).

Then, the Western blot was used to detect the levels of CD63, CD9, and Tsg101 proteins, which are commonly enriched and located in exosome membranes. As shown in Figure 1B, the three proteins could be detected in urinary exosomes from NSCLC patients and healthy controls. The full, uncropped, and unedited versions of Western blots are shown in Supplementary Figure S1. The result of NTA demonstrated that most of the exosomes had a diameter of approximately 143 ± 2.9 nm (Figure 1C).

### Differential expression analysis of lncRNAs

**Figure 2:**
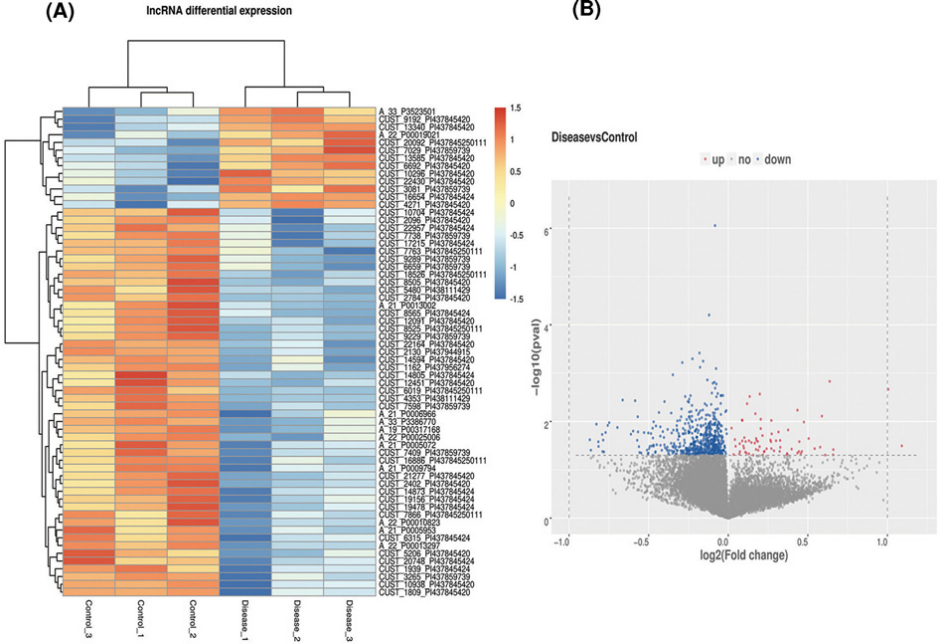
lncRNAs were differentially expressed in exosomes of NSCLC patients and control groups (**A**) Hierarchical clustering heatmaps showing lncRNA expression profiles (*P*<0.05). (**B**) Volcano plots showing all detected lncRNAs in urinary exosomes from NSCLC patients (*n*=3) and control groups (*n*=3).

Differential expression analysis between NSCLC patients (*n*=3) and healthy controls (*n*=3) was performed using microarrays. The results showed that the expression of 640 lncRNAs was significantly different between the two groups (Supplementary Table S2). Among them, 70 lncRNAs were up-regulated, while 570 were down-regulated (*P*<0.05). The top ten up- and down-regulated differentially expressed lncRNAs are listed in Table 3. The lncRNAs expression profile was established and clustered using hierarchical cluster analysis. The heatmap and volcano plots are shown in Figure 2A,B.

**Table 3:** Top ten up-regulated and ten down-regulated lncRNAs of urinary exosomes in NSCLC patients and controls

Probe name	Log2 fold change	Regulation	*P*_val_	Gene name	Chr
CUST_13585_PI437845420	0.64	Up	0.00	lnc-FRAT1-5	chr10
CUST_10296_PI437845420	0.59	Up	0.01	lnc-SRY-11	chrY
CUST_3081_PI437859739	0.48	Up	0.01	lnc-RNASE13-1	chr14
CUST_20092_PI437845250111	0.43	Up	0.01	CECR5-AS1	chr22
CUST_6692_PI437845420	0.20	Up	0.01	lnc-ANKIB1-2	chr7
CUST_9192_PI437845420	0.20	Up	0.00	lnc-GARNL3-2	chr9
CUST_7029_PI437859739	0.17	Up	0.01	lnc-EPHX3-3	chr19
CUST_13340_PI437845420	0.13	Up	0.00	lnc-KCNMB2-11	chr3
CUST_4271_PI437845420	0.12	Up	0.00	lnc-ITK-2	chr5
A_22_P00019021	0.09	Up	0.01	lnc-SETDB1-1:1	chr1
CUST_7763_PI437845250111	−0.26	Down	0.01	MIR143HG	chr5
CUST_7598_PI437859739	−0.27	Down	0.00	lnc-VRK3-1	chr19
CUST_2130_PI437944915	−0.29	Down	0.00	lnc-DPH2-1	chr1
CUST_10938_PI437845420	−0.29	Down	0.01	lnc-RP11-105C20.2.1-11	chr16
CUST_2096_PI437845420	−0.32	Down	0.01	lnc-PTTG2-3	chr4
A_22_P00013297	−0.35	Down	0.00	lnc-RP11-1105G2.3.1-2:1	chr12
CUST_2402_PI437845420	−0.36	Down	0.01	lnc-HELQ-1	chr4
CUST_3265_PI437859739	−0.40	Down	0.00	lnc-RP11-80A15.1.1-2	chr14
CUST_9229_PI437859739	−0.58	Down	0.00	lnc-ARL6IP6-4	chr2
CUST_1809_PI437845420	−0.66	Down	0.00	lnc-DGKQ-1	chr4

### Verification of differentially expressed lncRNAs in NSCLC patients

**Figure 3:**
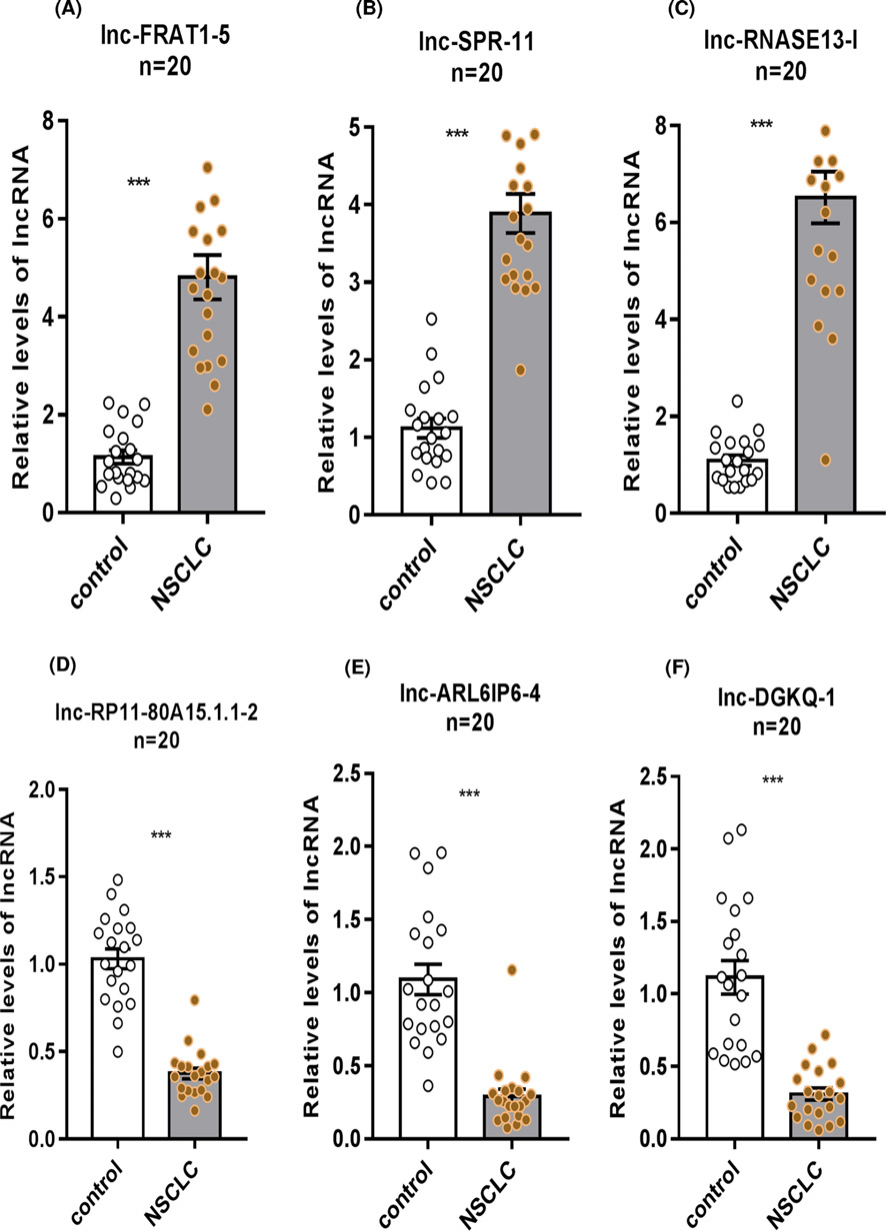
QT-PCR verification of differentially expressed lncRNAs Relative expression levels ofeach selected lncRNAs were verified in NSCLC patients (*n*=20) and the control group (*n*=20) using QT-PCR. (**A–C**) Expression levels of lnc-FRAT1-5, lnc-SRY-11, and lnc-RNASE13-1 in NSCLC patients were significantly higher than those in healthy individuals (mean ± SEM, ***P<0.001). (**D–F**) Expression levels of lnc-RP11-80A15.1.1-2, lnc-ARL6IP6-4, and lnc-DGKQ-1 were significantly down-regulated (mean ± SEM, ***P<0.001).

Three up-regulated and three down-regulated lncRNAs were chosen to be validated by QT-PCR. The data shown in Figure 3 verified that the expression levels of lnc-FRAT1-5, lnc-SRY-11, and lnc-RNASE13-1 in NSCLC patients (*n*=20) were significantly higher than those in healthy individuals (*n*=20) (*P<0.001*, Figure 3A–C). In turn, the expression levels of lnc-RP11-80A15.1.1-2, lnc-ARL6IP6-4, and lnc-DGKQ-1 were significantly down-regulated (*P<0.001*, Figure 3D–F). A melting curve of one of the primers is shown in Supplementary Figure S2.

### lncRNAs functional analysis by GO analysis and KEGG pathway

**Figure 4:**
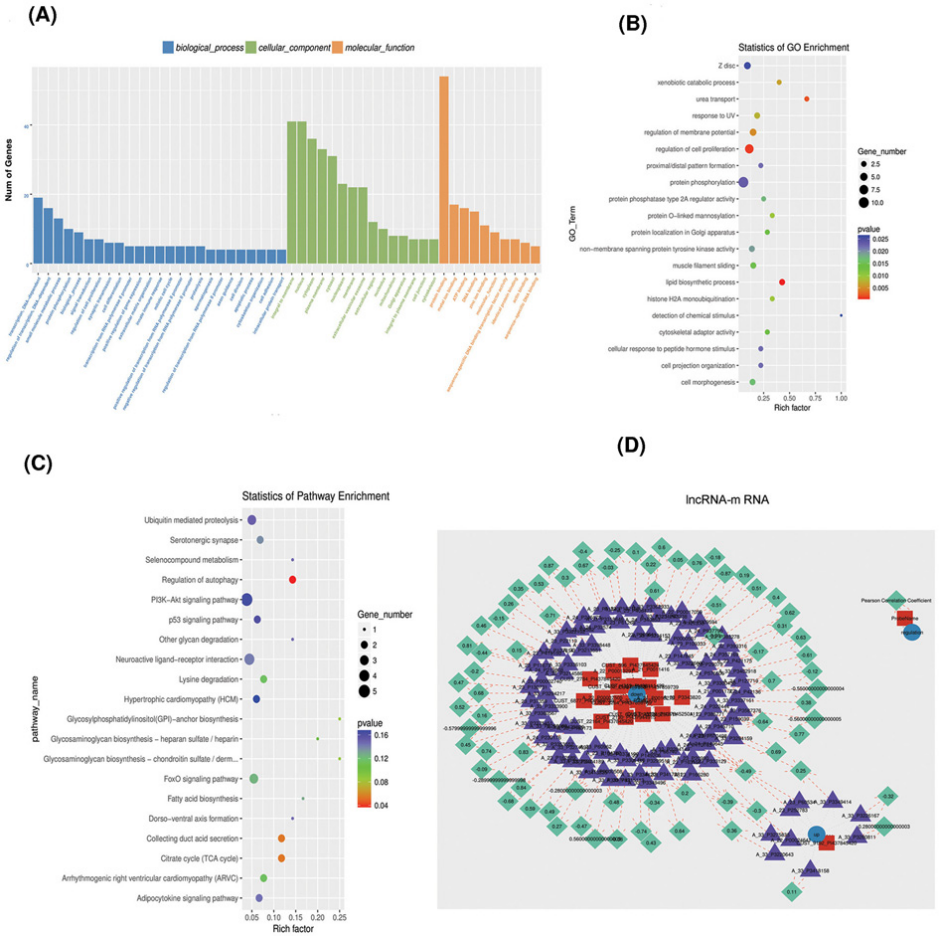
Functional and network analyses of lncRNAs (**A**) GO analysis for all lncRNA genes. Blue represents GO terms of biological processes, green represents the GO terms of cellular components, and orange represents GO terms of molecular functions. (**B**) GO enrichment histogram for differentially expressed lncRNA genes. (**C**) KEGG analysis based on lncRNA-target genes. (**D**) Construction of lncRNA–mRNA co-expression interaction network. A total of 98 lncRNAs and mRNAs were retained. Approx. 27 lncRNAs- mRNAs pairs (Pearson correlation ≥ 0.7 and ≤ −0.7) were selected to construct the lncRNA–mRNA network. Red square nodes represent 19 down-regulated lncRNAs, and purple triangle nodes represent mRNAs. Blue nodes represent regulation of mRNA. Green nodes represent the Pearson correlation coefficient.

GO analysis (biological processes, molecular functions, and cellular components) was used to analyze the differential expression of urinary lncRNAs between NSCLC patients and healthy controls (Figure 4A). The classification revealed the top 20 GO terms (Figure 4B). The KEGG pathway analysis identified associations with 20 pathways (*P<0.05*), including the PI3K-AKT signaling pathway, FOXO signaling pathway, fatty acid biosynthesis, and p53 signaling pathway (Figure 4C).

We used the TargetScan software for miRNA target prediction to theoretically predict interactions between lncRNAs and the *cis* and *trans* target miRNAs within the 100-kb window of each lncRNA (*P*≤*0.05*). The correlation between lncRNAs and mRNAs was expressed as an absolute value of the Pearson correlation ≥ 0.7 and ≤ −0.7. The data displayed each lncRNA and its potential complementary binding mRNAs (Figure 4D). A total of 98 lncRNAs and mRNAs were retained. Approximately 27 lncRNAs–mRNAs pairs (Pearson correlation ≥0.7 and ≤ −0.7) were selected to construct the lncRNA–mRNA network. The red square nodes in Figure 4D represent 19 down-regulated lncRNAs, while purple triangle nodes represent mRNAs, blue nodes represent the regulation of mRNA, and the green nodes represent the Pearson correlation coefficient.

## Discussion

The morbidity and mortality of lung cancer exhibits a significant rising trend worldwide [3], such that lung cancer has become a significant hazard to human health and survival. Therefore, the survival rate of lung cancer patients heavily depends on improving early diagnosis and timely, appropriate treatment [2].

As excellent biological material, urine samples can be acquired in large volumes and in an entirely noninvasive manner [31,32]. Urinary exosomes are a novel reservoir for biomarker discovery and possible mediators of intercellular signaling containing proteins, miRNAs, and lncRNAs secreted by various types of healthy or tumor cells in numerous biological functions [33]. Exosomes collectively convey a specific message for neighboring or distant cells [34]. They can also serve as carriers of bioactive molecules to promote tumorigenesis and tumor metastasis [35].

As mentioned in the literature review, lncRNA is a class of noncoding RNAs of >200-nt length that does not encode proteins. LncRNAs are known to be able to regulate gene expression through diverse mechanisms [36]. Abundant evidence indicates that alterations in the expression profile of lncRNAs are associated with cancer tumorigenesis, invasion, differentiation, and metastasis, and may therefore serve as early disease indicators [37,38]. Available data indicate that numerous lncRNA levels are aberrant in NSCLC, and these lncRNAs play crucial roles in regulating NSCLC-associated pathways. Furthermore, they play very important roles in the critical biological process of NSCLC, such as tumor growth, metastasis, and angiogenesis [39,40].

Recently, some definite lncRNAs were discovered in exosomes secreted from lung cancer cells. Tang et al. reported that lncRNA *AFAP1-AS1* overexpression significantly promoted NSCLC cell proliferation, migration, and invasion, while inhibiting apoptosis in NSCLC [41]. Furthermore, the binding of *AFAP1-AS1* to IRF7 leads to the activation of the RIG-I like receptor signaling pathway and BclAS12, which may result in NSCLC proliferation and progression. These findings provide insight into a novel therapeutic target for NSCLC.

The present study reveals hundreds of lncRNAs and miRNAs. A total of 640 differentially expressed lncRNAs in exosomes secreted from the urine of the NSCLC patients were screened in comparison with those of healthy controls. Among them, 70 lncRNAs were significantly up-regulated, while 570 lncRNAs were significantly down-regulated. According to the QT-PCR results, lnc-FRAT1-5, lnc-SRY-11, and lnc-RNASE13-1 were up-regulated; lnc-RP11-80A15.1.1-2, lnc-ARL6IP6-4, and lnc-DGKQ-1were down-regulated.

LncRNAs could interact with mRNAs to modulate the biological characteristics of NSCLC [42]. We predicted the potential target genes and constructed the co-expression network to elucidate the underlying molecular mechanisms of lncRNA function, which indicates that several lncRNAs may be involved in the pathogenesis of NSCLC. The results demonstrate actual differences in lncRNA expression of urinary exosomes between NSCLC patients and healthy controls.

The GO-enriched cluster analysis in the present study shows that in the cellular component class, the putative target genes are mostly related to the integral membrane, nucleus, and cytoplasm. In the biological process class, the putative target genes were mainly associated with transcription, small molecule metabolic process, and protein phosphorylation. In the molecular function class, the putative target genes were mostly related to the binding of protein, metal ions, and ATP.

The KEGG pathway analysis in the present study reveals that these target genes are significantly enriched in 20 pathways. The KEGG pathways were mostly enriched in the PI3K-AKT, FOXO, and p53 signaling pathways, as well as fatty acid biosynthesis. The differentially expressed lncRNAs in our study may regulate these pathways, which are associated with tumor cell proliferation, tumor cell apoptosis, and the cell cycle as part of their involvement in the pathogenesis of NSCLC.

Numerous studies showed that the PI3K/AKT/mTOR signaling pathway activates upstream receptors (EGFR and PDGF) and is mutated in a variety of cancers, including breast cancer, gastric cancer, and NSCLC [43–45]. The PI3K/Akt and p53 signaling pathways play a pivotal role in the development of NSCLC, regulating cell survival, proliferation, anti-apoptosis, and angiogenesis [46,47]. Dysregulation of the PI3K/AKT pathway has been reported to frequently occur in NSCLC [48]. Furthermore, the FOXO signaling pathway is closely associated with the PI3K/Akt signaling pathway.

Although we obtained positive results, it is necessary to further clarify the specific role of these lncRNAs in NSCLC. Furthermore, in future studies, we will explore the biological functions, mechanism of action, and signaling pathways of the differential expressed urinary exosome lncRNAs *in vitro*. In particular, we will focus on whether they are associated with the PI3K/AKT, FOXO, and p53 signaling pathways, fatty acid biosynthesis, and the p53 signaling pathway.

A shortcoming of our study is that we did not carry out a validation process. Further studies must be performed to reveal the role and regulatory mechanisms underlying the lncRNAs–mRNA network, and to determine whether the expression level of these lncRNAs has already been altered at the very early stages of NSCLC. Furthermore, whether the expression level of these lncRNAs is changed before and after treatment remains a topic for future discussion.

In summary, lnc-FRAT1-5, lnc-SRY-11, lnc-RNASE13-1, lnc-RP11-80A15.1.1-2, lnc-ARL6IP6-4, and lnc-DGKQ-1 may be important regulators of NSCLC. Moreover, the present results suggest that these lncRNAs may regulate the PI3K/Akt, FOXO, or p53 signaling pathways. It was also demonstrated that these six lncRNAs may serve as potential markers of NSCLC metastasis.

## Supplementary Material

Supplementary Figures S1-S2 and Tables S1-S2

## Data Availability

The data included in the current study are available from the Refseq (https://www.ncbi.nlm.nih.gov/refseq/), Ensembl (http://www.ensembl.org/), UCSC (https://genome.UCSC.edu/), GENCODE (https://www.gencodegenes.org/), NONCODE (http://www.noncode.org), and LNCipedia (http://www.lncipedia.org/) databases.

## Abbreviations

GO: gene ontology
HRP: horse radish peroxidase
KEGG: Kyoto Encyclopedia of Genes and Genomes
lncRNA: long noncoding RNA
NSCLC: non-small cell lung cancer
NTA: nanoparticle tracking analysis
QT-PCR: quantitative real-time PCR
