# Supplementary material for: Urinary exosomal long noncoding RNAs serve as biomarkers for early detection of non-small cell lung cancer
Source: Biosci Rep. 2021 Oct 12;41(10):BSR20210908. doi: 10.1042/BSR20210908 (PMC8521532; doi:10.1042/BSR20210908)
Supplement: Supplementary Figures S1-S2 and Tables S1-S2 [file BSR-2021-0908_supp.pdf]

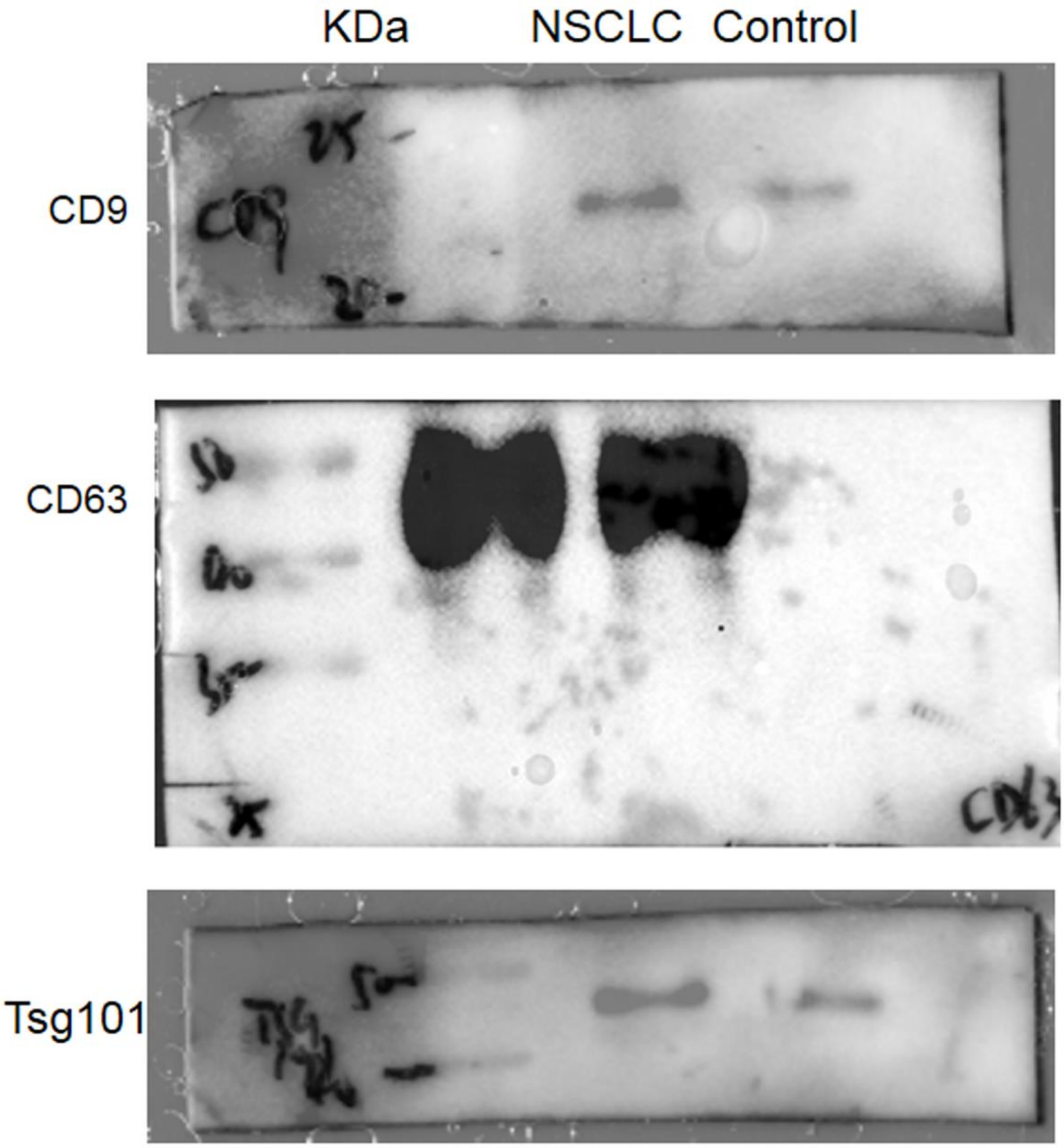

Figure S1. Western blot results of CD63, CD9,andTsg101. Full uncropped and unedited versions of the western blots. Lane 1: protein ladder, Lane 2: NSCLC patient sample, Lane 3: healthy control sample.

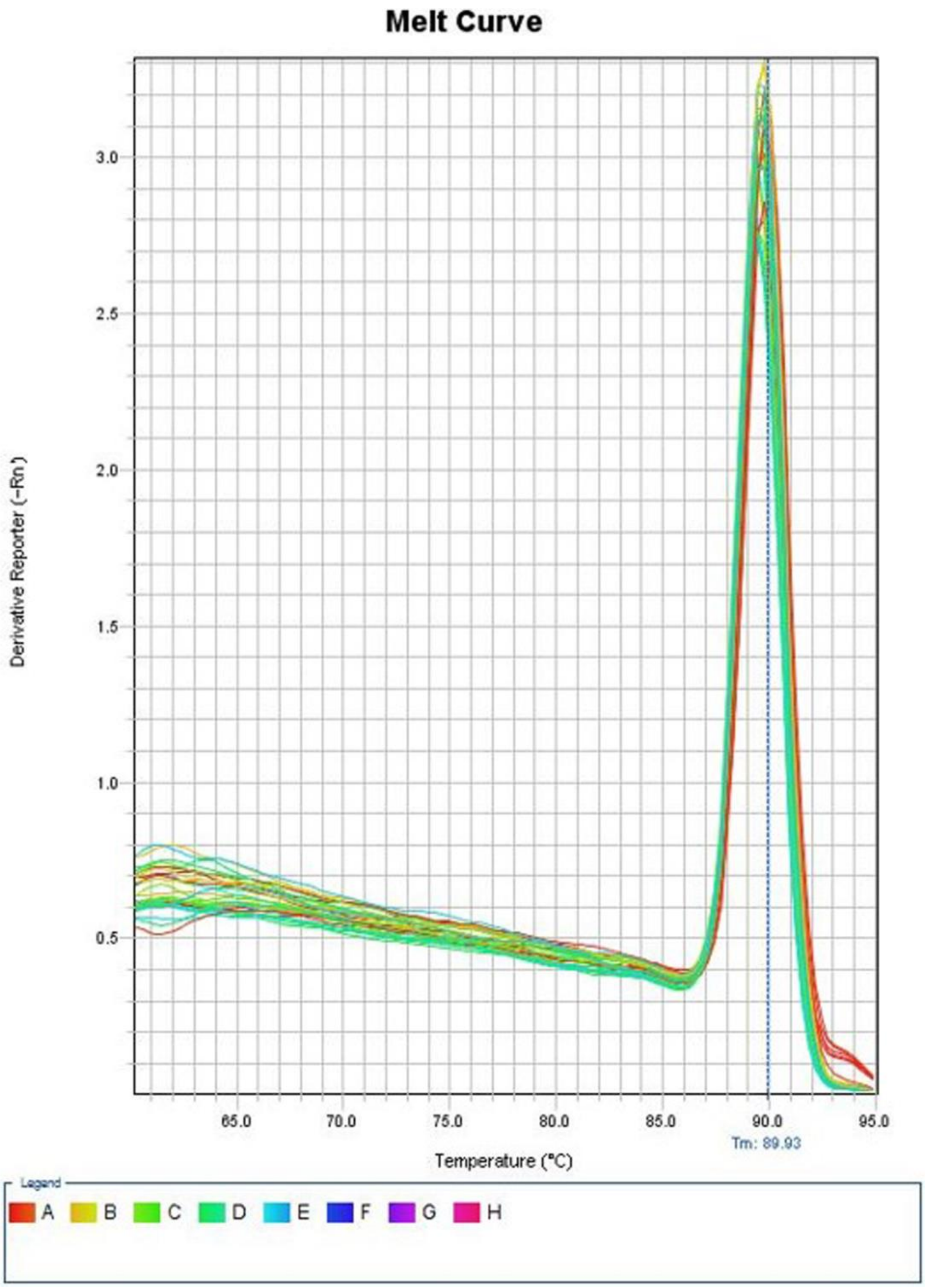

Figure S2. Melting Curve of lnc-FRAT1-5. There is no impurity peak in the melting curve.

Table S1. Clinical characteristics ofNSCLC patients and healthy controls of Figure 2.

Study groups	Gender	Age	Pathological type	Clinical stage
**NSCLC patients**	Male	68	Squamouscarcinoma	I+II stage
**NSCLC patients**	Male	61	Adenocarcinoma	III+IV stage
**NSCLC patients**	Female	65	Adenocarcinoma	I+II stage
**healthy controls**	Male	52		
**healthy controls**	Male	59		
**healthy controls**	Female	61		

Table S2. 100 Differencial expression lncRNAs of urinary exosomes in NSCLC patients and controls.

ProbeName	log2FoldChange	regulation	pval	GeneName	Chr
CUST_3264_PI437859739	-0.08	down	0.00	lnc-SDR39U1-1	chr14
A_33_P3329462	-0.12	down	0.00	DLEU1 antisense RNA 1	chr13
CUST_4353_PI438111429	-0.18	down	0.00	PAOX	chr10
CUST_2784_PI437845420	-0.22	down	0.00	lnc-TMEM184C-5	chr4
CUST_6019_PI437845250111	-0.16	down	0.00	LINC00969	chr3
CUST_2130_PI437944915	-0.29	down	0.00	lnc-DPH2-1	chr1
CUST_18526_PI437845250111	-0.18	down	0.00	CTD-2528A14.1	chr19
A_21_P0008815	-0.08	down	0.00	lnc-C15orf2-1:1	chr15
A_22_P00013297	-0.35	down	0.00	lnc-RP11-1105G2.3.1-2:1	chr12
CUST_22164_PI437845420	-0.13	down	0.00		chr10
CUST_13585_PI437845420	0.64	up	0.00	lnc-FRAT1-5	chr10
CUST_2587_PI437859739	-0.09	down	0.00	lnc-TRPC4-2	chr13
A_22_P00007505	-0.10	down	0.00	lnc-GYPA-1:1	chr4
A_21_P0010215	-0.07	down	0.00	uncharacterized LOC101928576	chr21
A_21_P0011416	-0.08	down	0.00		chr16
CUST_16654_PI437845424	1.01	up	0.00		chr5
CUST_10328_PI437845424	-0.10	down	0.00		chr2
A_33_P3343820	-0.07	down	0.00	long intergenic non-protein coding RNA 632	chrX
CUST_9192_PI437845420	0.20	up	0.00	lnc-GARNL3-2	chr9
CUST_1452_PI437859739	-0.03	down	0.00	lnc-HDAC7-1	chr12
CUST_596_PI437845424	-0.04	down	0.00		chr11
CUST_2235_PI437845424	-0.05	down	0.00		chr12
A_33_P3386770	-0.24	down	0.00	lnc-MAPK15-3:3	chr8
CUST_13340_PI437845420	0.13	up	0.00	lnc-KCNMB2-11	chr3
CUST_7598_PI437859739	-0.27	down	0.00	lnc-VRK3-1	chr19
CUST_6877_PI437859739	-0.07	down	0.00	lnc-ELAVL1-1	chr19
CUST_21277_PI437845420	-0.25	down	0.00		chr1
CUST_1809_PI437845420	-0.66	down	0.00	lnc-DGKQ-1	chr4
CUST_9229_PI437859739	-0.58	down	0.00	lnc-ARL6IP6-4	chr2
CUST_1162_PI437956274	-0.25	down	0.00	TMPRSS4	chr11
CUST_3265_PI437859739	-0.40	down	0.00	lnc-RP11-80A15.1.1-2	chr14
CUST_8505_PI437845420	-0.25	down	0.00	lnc-C9ORF123-7	chr9
CUST_625_PI437944915	-0.05	down	0.00	PRR36	chr19
CUST_4271_PI437845420	0.12	up	0.00	lnc-ITK-2	chr5
A_19_P00317168	-0.15	down	0.00		chr10
CUST_6705_PI437845420	-0.11	down	0.00	lnc-GNGT1-2	chr7
CUST_7409_PI437859739	-0.23	down	0.00	lnc-PRR19-1	chr19
CUST_5480_PI438111429	-0.14	down	0.00	lnc-RP11-664I21.6.1-1	chr11

CUST_2899_PI437944915	-0.07	down	0.01	lnc-LYSMD1-3	chr1
CUST_10938_PI437845420	-0.29	down	0.01	lnc-RP11-105C20.2.1-11	chr16
CUST_19112_PI437845420	-0.08	down	0.01	CAHM	chr6
CUST_8525_PI437845250111	-0.16	down	0.01	BVES-AS1	chr6
CUST_20092_PI437845250111	0.43	up	0.01	CECR5-AS1	chr22
CUST_14594_PI437845420	-0.20	down	0.01	lnc-TMCO5A-3	chr15
CUST_22841_PI437845250111	-0.01	down	0.01	linc-FAM153A-2	chr5
A_22_P00025006	-0.17	down	0.01	lnc-SHISA9-4:1	chr16
A_22_P00010823	-0.23	down	0.01	long intergenic non-protein coding RNA 1470	chr5
CUST_17296_PI437845250111	-0.02	down	0.01	RP11-156P1.3	chr17
CUST_19478_PI437845424	-0.25	down	0.01		chr7
CUST_17215_PI437845424	-0.44	down	0.01		chr6
A_21_P0000741	-0.10	down	0.01	ADAMTS9 antisense RNA 2	chr3
CUST_22957_PI437845424	-0.18	down	0.01		chr9
CUST_2402_PI437845420	-0.36	down	0.01	lnc-HELQ-1	chr4
A_21_P0011909	-0.08	down	0.01		chr2
CUST_7029_PI437859739	0.17	up	0.01	lnc-EPHX3-3	chr19
A_19_P00807507	-0.05	down	0.01	lnc-MRGPRF-4:2	chr11
CUST_1939_PI437845424	-0.19	down	0.01		chr12
A_21_P0005953	-0.13	down	0.01	lnc-KHDRBS3-5:4	chr8
CUST_5206_PI437845420	-0.14	down	0.01	lnc-YIPF3-2	chr6
CUST_17529_PI437845420	-0.12	down	0.01	lnc-TEC-2	chr4
A_21_P0012140	-0.09	down	0.01	uncharacterized LOC101926935	chr20
CUST_10296_PI437845420	0.59	up	0.01	lnc-SRY-11	chrY
CUST_16886_PI437845250111	-0.23	down	0.01	AL353997.3	chr17
CUST_20748_PI437845424	-0.24	down	0.01		chr8
CUST_8565_PI437845424	-0.57	down	0.01		chr2
CUST_10704_PI437845424	-0.30	down	0.01		chr20
CUST_11506_PI437845424	-0.09	down	0.01		chr21
CUST_6659_PI437859739	-0.25	down	0.01	lnc-POLR2E-2	chr19
CUST_6315_PI437845424	-0.17	down	0.01		chr17
CUST_12960_PI437845250111	-0.09	down	0.01	RP11-831A10.2	chr11
CUST_14229_PI437845250111	-0.08	down	0.01	RP11-394A14.4	chr13
CUST_19156_PI437845424	-0.18	down	0.01		chr7
CUST_9289_PI437859739	-0.24	down	0.01	lnc-PSMD14-4	chr2
CUST_14873_PI437845424	-0.14	down	0.01		chr4
CUST_6692_PI437845420	0.20	up	0.01	lnc-ANKIB1-2	chr7
CUST_7763_PI437845250111	-0.26	down	0.01	MIR143HG	chr5
A_22_P00016230	-0.07	down	0.01	CATIP antisense RNA 1	chr2
CUST_12091_PI437845420	-0.20	down	0.01	lnc-ARNTL-4	chr11

A_33_P3523501	0.09	up	0.01		chr19
CUST_14805_PI437845424	-0.30	down	0.01		chr4
A_22_P00019021	0.09	up	0.01	lnc-SETDB1-1:1	chr1
CUST_22430_PI437845420	0.27	up	0.01		chr10
CUST_2096_PI437845420	-0.32	down	0.01	lnc-PTTG2-3	chr4
CUST_7738_PI437859739	-0.23	down	0.01	lnc-FAM71E2-1	chr19
A_21_P0005072	-0.23	down	0.01	lnc-NOX3-2:1	chr6
A_21_P0013669	-0.11	down	0.01	uncharacterized LOC101927993	
A_21_P0013002	-0.47	down	0.01		chr5
CUST_1895_PI437845420	-0.06	down	0.01	lnc-CYTL1-2	chr4
CUST_3081_PI437859739	0.48	up	0.01	lnc-RNASE13-1	chr14
CUST_12451_PI437845420	-0.75	down	0.01	lnc-FAM69C-4	chr18
CUST_7974_PI437845424	-0.08	down	0.01		chr19
CUST_8082_PI437845250111	-0.12	down	0.01	ELOVL2-AS1	chr6
CUST_2030_PI437944915	-0.06	down	0.01	lnc-SNIP1-1	chr1
A_21_P0009794	-0.25	down	0.01	lnc-VN1R2-1:2	chr19
CUST_14622_PI437845424	-0.09	down	0.01		
A_21_P0006966	-0.27	down	0.01	uncharacterized LOC101928322	chr10
A_22_P00003087	-0.12	down	0.01	lnc-C8orf74-1:1	chr8
CUST_449_PI437944915	-0.12	down	0.01	PNMT	chr17
CUST_7866_PI437845250111	-0.14	down	0.01	CTB-174D11.1	chr5
